# Suicidal Behavior in Fibromyalgia Patients: Rates and Determinants of Suicide Ideation, Risk, Suicide, and Suicidal Attempts—A Systematic Review of the Literature and Meta-Analysis of Over 390,000 Fibromyalgia Patients

**DOI:** 10.3389/fpsyt.2021.629417

**Published:** 2021-11-19

**Authors:** Mohammad Adawi, Wen Chen, Nicola Luigi Bragazzi, Abdulla Watad, Dennis McGonagle, Yarden Yavne, Adi Kidron, Hadas Hodadov, Daniela Amital, Howard Amital

**Affiliations:** ^1^Padeh and Ziv Medical Centers, Azrieli Faculty of Medicine, Bar-Ilan University, Safed, Israel; ^2^Department of Psychiatry, Xiamen Xianyue Hospital, Xiamen, China; ^3^Department of Health Sciences (Dipartimento di Scienze della Salute), Postgraduate School of Public Health, University of Genoa, Genoa, Italy; ^4^Laboratory for Industrial and Applied Mathematics (LIAM), Department of Mathematics and Statistics, York University, Toronto, ON, Canada; ^5^Section of Musculoskeletal Disease, NIHR Leeds Musculoskeletal Biomedical Research Unit, Leeds Institute of Molecular Medicine, Chapel Allerton Hospital, University of Leeds, Leeds, United Kingdom; ^6^Department of Medicine 'B', Sheba Medical Center, Tel HaShomer, Israel; ^7^Zabludowicz Center for Autoimmune Diseases, Sheba Medical Center, Tel Hashomer, Israel; ^8^Sackler Faculty of Medicine, Tel Aviv University, Tel Aviv, Israel; ^9^Ness Ziona Beer-Yaakov Mental Health Center, Beer Yaakov, Israel

**Keywords:** fibromyalgia, suicidal ideation and attempt, PRISMA guidelines, systematic review, meta analysis

## Abstract

**Background:** Suicide is a leading cause of death worldwide, affecting ~800,000 people every year. Fibromyalgia is an extremely prevalent rheumatic disease with a predisposition for comorbid anxiety and depression, which are known risk factors for suicidal behavior. Suicidality and relevant risk factors for suicidal behavior have not been thoroughly studied in patients with fibromyalgia.

**Objectives:** To investigate the risk of suicidal ideation and attempts in patients with fibromyalgia.

**Methods:** A systematic review and meta-analysis was conducted and reported according to the “Preferred Reporting Items for Systematic reviews and Meta-analyses” (PRISMA) standards. Also, the gray literature was extensively searched.

**Results:** Thirteen studies were included in the present systematic review and meta-analysis, including 394,087 fibromyalgia patients. Sample size ranged from 44 to 199,739 subjects, mean age ranged from 45.8 to 54.5 years while the female percentage with fibromyalgia ranged from 17.1 to 100.0%. The overall suicide ideation prevalence was 29.57% (95%CI 1.84–72.07), with an OR 9.12 of (95%CI 1.42–58.77), ranging from 2.34 (95%CI 1.49–3.66) to 26.89 (95%CI 5.72–126.42). Pooled suicide attempt prevalence was 5.69% [95%CI 1.26–31.34], with an OR of 3.12 [95%CI 1.37–7.12]. Suicide risk was higher with respect to the general population with an OR of 36.77 (95%CI 15.55–96.94), as well as suicide events with an HR of 1.38 (95%CI 1.17–1.71). Determinants of suicidality were found to be: employment status, disease severity, obesity and drug dependence, chronic pain and co-morbidities, in particular depression, anxiety, poor sleep, and global mental health. However, in some cases, after adjusting for psychiatric conditions, the threshold of statistical significance was not achieved.

**Conclusion:** Fibromyalgia patients are particularly prone to suicide, in terms of ideation, attempt, risk and events, warranting a pre-emptive screening of their mental health status. Given the few studies available, the high amount of heterogeneity, the evidence of publications bias and the lack of statistical significance when adjusting for underlying psychiatric co-morbidities, further high-quality studies should be conducted.

**Clinical Trial Registration:** ClinicalTrial.gov, identifier 10.17605/OSF.IO/Y4BUE.

## Introduction

Suicide is a leading cause of mortality worldwide, accounting for 1.4% of premature deaths. According to the World Health Organization (WHO), in 2015, ~800,000 deaths were due to suicide, roughly estimating as one person every 40 seconds (1). Suicide is a complex, multi-factorial phenomenon, deriving from the interaction between individual characteristics and environmental factors. Suicide attempts and ideation may be antecedents of suicide and as such, represent important predictors of suicidal behaviors (2). Suicidality includes a diverse and dynamic spectrum, ranging from death wishes and suicidal thoughts to suicide attempts and completed suicides.

Most suicidal behaviors and suicides occur in patients with underlying psychiatric diseases (1), including depression and mood disorders (3), substance abuse and dependence (4), schizophrenia and other psychoses (5), anxiety (6), personality disorders (7), eating disorders (8) and trauma-related disorders (9). Co-occurrence of suicide and organic mental disorders which are characterized by neurological impairment and dysfunction, such as multiple sclerosis (10) and epilepsy (11), is also frequently reported.

Among physical illnesses, chronic conditions such as asthma (12) and renal failure (13), significantly increase the risk of suicidality (1). Fibromyalgia is a chronic condition, characterized by widespread, musculoskeletal pain, morning stiffness, hypersensitivity to physical and physiological stimuli, sleep disorders and prominent fatigue. It is the second most common musculoskeletal condition, affecting 0.2–6.6% of the population worldwide (14), with a higher prevalence rate among women (in the range 2.4–6.8%) (15). As a chronic pain disorder, fibromyalgia patients suffer from high rates of comorbid anxiety and depression, which, as previously mentioned, are well-known risk factors for suicidal behaviors (16–18).

Suicidality has been well-studied with relation to other chronic pain conditions, however there is a dearth of information with regard to suicidality amongst fibromyalgia patients. Although several large-scale studies have reported a statistically significant increased risk of suicide among fibromyalgia patients, these findings were not replicated in subsequent studies. Therefore, we conducted the present systematic review and meta-analysis with the aim of evaluating the risk of suicidal ideation, behavior, attempts and events amongst patients with fibromyalgia.

## Materials and Methods

### Study Protocol and Design

The study protocol of the present systematic review and meta-analysis was conducted in accordance with the “Preferred Reporting Items for Systematic reviews and Meta-Analyses–Protocol” (PRISMA-P) guidelines (19). The study protocol is available upon formal request to the Corresponding Authors. It has been registered in the “Open Science Framework” (OSF) database (Registration Code 10.17605/OSF.IO/Y4BUE).

### Research Team

The team involved in the process was multi-disciplinary and comprised of several members, including an expert of research methodology, biostatistics, and epidemiology (NLB), experts in the field of psychology and psychiatry (WC, NLB, HD, DA), experts in the field of rheumatology (AW, HA, DMG, and MA) and experts in the field of internal medicine (HA).

### Review Aim and Purposes

The research question was generated after an extensive consultation of the research team. The review questions were: (i) What are the suicidal ideation, attempt and event rates among fibromyalgia patients? (ii) What are the main determinants of suicidal behavior among fibromyalgia patients?

The study's aim was to synthesize the existing scholarly literature concerning suicide rate among fibromyalgia patients and its determinants. Findings were presented by means of charts, tables and figures, together with a detailed, narrative report of the literature.

### Literature Search

Several scholarly databases, including PubMed/MEDLINE, Scopus, ISI/Web of Science (WoS), were searched, using a string of proper keywords, such as “fibromyalgia” and “suicide” (and synonyms), connected by Boolean operators. Wild card option (truncated keywords) and Medical Subject Headings (MeSH) terms were used where appropriate. Research strategy was adapted for each database, modifying the string of keywords accordingly. Gray literature was mined performing an extensive search of Google Scholar and Directory of Open Access Journals (DOAJ). Conference abstracts and proceedings were assessed as well. Relevant available reviews on the study topic were not included in the present systematic review and meta-analysis, yet were thoroughly evaluated with the goal of discovering additional potentially eligible studies. Further details about search strategy are reported in Supplementary Table 1.

Reference lists of potentially eligible studies were consulted in order to reduce the risk of missing relevant articles. Furthermore, target journals were hand-searched for potentially relevant studies. Artificial Intelligence techniques (such as Natural Language Processing (NLP)-based approaches) were utilized to facilitate and aid screening and selection process.

Literature search was carried out on May 31, 2019.

### Inclusion Criteria

- Studies meeting the following PICOS criteria were considered for inclusion:
- P (patient, problem or population): fibromyalgia patients;
- I/E (intervention/exposure or phenomenon of interest): suicidality/suicidal behavior (in terms of suicide risk, suicidal ideation, attempt and event);
- C (comparison, control or comparator): any comparator (fibromyalgia patients *vs*. general population or patients suffering from other diseases);
- (outcome/outcomes of interest): prevalence/incidence and determinants of suicidality/suicidal behavior among fibromyalgia patients, within the conceptual framework of the “ideality to action” framework; and
- Study design/characteristics: original observational articles, prevalence/incidence studies.

Furthermore, the following criteria were taken into consideration:

- Time: no time filter/restraint (scholarly databases searched from inception); and
- Languages: no language filter/restraint (that is to say, all the full complement of languages available). In case of inclusion of non-English articles, they were acquired in full-text and translated by expert translators with expertise in the field of medicine and related health-allied disciplines.
- The reader is referred to Supplementary Table 1 for further details.

### Exclusion Criteria

Studies not meeting the above-mentioned PICOS criteria were excluded (Supplementary Table 1). More in detail, exclusion criteria were:

- P: patients with fibromyalgia as co-morbidity and not as the main diagnosis;
- I/E: fibromyalgia patients without suicidal behavior;
- C: cases vs. controls;
- O: studies not reporting outcome(s) of interest or with insufficient details to compute them; and
- S: review articles, letter to editor, editorial, expert opinion, commentary, clinical case reports or series; and interventional studies (including clinical trials).

Studies excluded with reasons were noted and reported (Table 1).

**Table 1:** List of studies excluded with reasons.

**Study excluded with reason**	**Reason for exclusion**
Amir et al. (17)	Not meeting inclusion/exclusion criteria
Liu et al. (20)	Not meeting PICOS criteria (FM as a co-morbidity and not as the main diagnosis)

*FM, fibromyalgia*.

### Data Extraction

The following data were retrieved: namely, study publication year, country, sample size, and main characteristics of the recruited sample (age, female percentage, educational level, employment and marital status, and disease activity). Data were extracted independently by two reviewers. Data extraction was pilot-tested on a sample of five articles randomly selected from the pool of included studies. In case of disagreement between reviewers, a third author was consulted and acted as a final referee. For further details, the reader is referred to Supplementary Table 2.

### Study Appraisal

Methodological quality of the included studies was critically appraised using the Newcastle-Ottawa scale (NOS) (21). This tool explores three dimensions: namely, study selection (in terms of representativeness of the sample and the reliability of its recruitment method), comparability of the recruited sample with other studies in literature, and outcome(s) (in terms of its/their assessment, adequate follow-up, and sufficient follow-up duration).

### Meta-Analysis

Meta-analysis was conducted using the commercial software “Comprehensive Meta-analysis” (CMA version 3.0) and MedCalc Statistical Software version 16.8.4 (MedCalc Software bvba, Ostend, Belgium; https://www.medcalc.org; 2016). Rates were pooled together computing the effect size (ES) with its 95% confidence interval (CI). For prevalence figures, the logit transformation approach was utilized in the current meta-analysis, being one of the possible approaches for pooling together raw prevalence data. The following equation was used to compute the *logit* transformation *l*:


$$\begin{array}{l} l=\ln\left(\frac{p}{1-p}\right) \end{array}$$

where *p* is the prevalence proportion.

Variance was computed using the following equation:


$$\begin{array}{l} Var\left(l\right)=\frac{1}{N•p}+\frac{1}{N•\left(1-p\right)} \end{array}$$

where *N* is the study population size.

The pooled *logit* transformation was subsequently back-transformed to a proportion utilizing the following equation:


$$\begin{array}{l} p=\frac{e^{l}}{e^{l}+1} \end{array}$$

Heterogeneity among studies was quantified carrying out the *I*^2^ and Q statistics. If *I*^2^ > 50.0%, heterogeneity was considered statistically significant and the random-effect model was utilized instead of the fixed-effect model. To investigate the sources of heterogeneity, meta-regression analyses (both uni-variate and multi-variate) were carried out, based on study publication year, country, sample size, quality, and main characteristics of the recruited sample (age, female percentage, educational level, employment and marital status, and disease activity) as moderators. In order to assess the stability and reliability of our findings, cumulative analysis was performed, removing one study per time and verifying the impact on the pooled ES. Sensitivity analysis based on study publication year, country, sample size and quality was performed. Publication bias was studied visually inspecting the funnel plot and carrying out the Egger's regression test. In case of the presence of publication bias, the “true” ES was estimated using the Duval and Tweedie's trim-and-fill method.

All figures with *p* < 0.05 were considered statistically significant.

## Results

### Systematic Review

**Figure 1:**
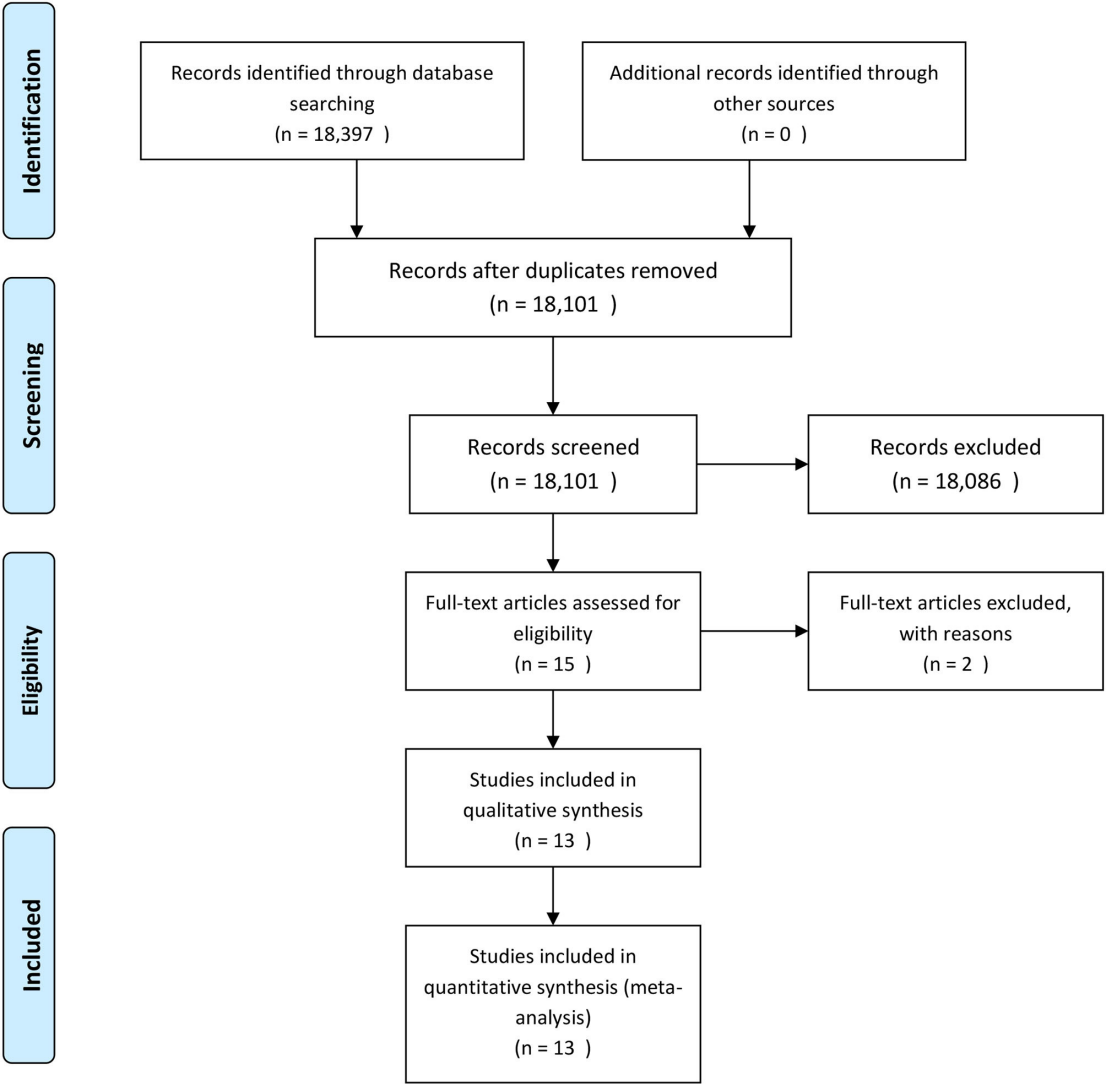
The process of study retrieval, selection and inclusion adopted in the present systematic review and meta-analysis.

As pictorially shown in Figure 1, the initial search yielded a pool of 18,397 items. After removing duplicates, 18,101 studies were screened for potential inclusions. Studies were excluded in accordance with the exclusion criteria reported in Supplementary Table 1 (17, 22).

Finally, 13 studies (20, 23–34) (Table 2 and Supplementary Table 3) have been included in the present systematic review and meta-analysis, investigating a total sample of 394,087 fibromyalgia patients. Sample size ranged from 44 (29) to 199,739 (24) subjects: mean age ranged from 45.8 (32) to 54.5 (29) years while female percentage ranged from 17.1 (29) to 100.0% (20). Marital status ranged from 38.6 (30) to 86.3% (31). The percentage of subjects who had completed primary school ranged from 38.6 (29) to 61.5% (31), while the unemployment rate ranged from 5.6 (31) to 13.6% (26). Five and four studies were carried out in Spain (26, 29–31, 33), and in the USA (24, 27, 28, 34), respectively, whereas the remaining four studies were performed in Denmark (25), Canada (23), Taiwan (32) and Israel (20). Seven studies (23–25, 27, 28, 32, 34) were database-based, while three (26, 30, 31) and three (20, 29, 33) were cross-sectional and case-control studies, respectively. No longitudinal studies could be found. The diagnosis of fibromyalgia was mostly based on the 1999 ACR criteria, with the exception of two studies, one of which utilized the 2010 ACR criteria (27), and the second (34) which utilized machine learning techniques, and combined diagnostic criteria, clinical expertise and textual information from clinical charts and medical records. The diagnosis of suicidality was made by asking *ad hoc* questions in two studies (23, 26), or utilizing structured, validated, and reliable psychometric tools in seven studies (20, 25, 26, 29–31, 33). One study (24) did not report sufficient details about methodology adopted, while one study (34), as also previously mentioned for the diagnosis of fibromyalgia, deployed an ensemble of techniques, combining machine learning approaches, literature review and clinical expertise. Three studies (27, 28, 32) evaluated suicide event (categorized in completed suicide, suicide attempt, and non-suicidal self-inflicted injury).

**Table 2:** Main characteristics of included studies.

**Authors**	**Year**	**Country**	**Study design**	**Sample size**	**Gender**	**Age**	**Suicidal variable studied**	**Epidemiological findings**	**Main findings**
Amir et al. (20)	2000	Israel	Cross-sectional, case-control study	51 outpatients randomly chosen from 600	F 100%	48.96 ± 8.41	Suicide risk	44.5 ± 8.4 SRS score	No differences in terms of suicide risk with general population and other patients (RA, LBP)
Ratcliffe et al. (23)	2008	Canada	Retrospective database-based study (data drawn from the Canadian Community Health Survey Cycle 1.2 file) study	595	NR	NR	SI, SA	OR ranging from 1.21 [95%CI 0.68–2.18] to 2.34 [95%CI 1.49–3.66] for SI, OR ranging from 1.27 [95%CI 0.54–3.01] to 3.12 [95%CI 1.37–7.12] for SA	No significant SI and SA OR after adjusting for psychiatric conditions and/or other co-morbid chronic pan conditions
Cheng et al. (24)	2009	USA	Retrospective database-based study	199,739	NR	NR	Suicide risk	Incidence 31/100,000 person/years for SB, OR 3.5 for SB	Increased suicide risk among FM patients
Dreyer et al. (25)	2010	Denmark	Retrospective-prospective database-based study (data drawn from the hospital database and from the Danish Mortality Register)	1,269 (1,353 out of an initial list of 1,361)	F 93.2%	58.2% in the range 30–49 years	Suicide event	8 suicide events (SMR 10.5 [95%CI 4.5–20.7] for all patients, 4 suicide events (SMR 6.5 [95%CI 1.8–16.7] for confirmed FM patients, 2 suicide events (SMR 19.6 [95%CI 2.2–70.8] for possible FM patients	Increased suicide risk at the time of diagnosis until 5 years after the diagnosis
Calandre et al. (26)	2011	Spain	Cross-sectional study	180 out of 795 (completion rate of 22.6%)	F 97.8%	51 ± 8.5 [22–74]	SA	30 (16.7%) for SA (21 (70%) by drug poisoning); 20 (66.7%) one SA, 5 (16.7%) two SA and 5 (16.7%) reported three SA	Higher SA rate with respect to general population
Wolfe et al. (27)	2011	USA	Retrospective database-based study (data drawn from the Wichita nonparticipant group file and from the NDB)	8,186	F 93.9%	50.5 ± 12.4	Suicide risk	OR 3.31 [95%CI 2.15–5.11] with respect to general population	Increased suicide risk
Ilgen et al. (28)	2013	USA	Retrospective database-based study (data drawn from the VA National Patient Care Database and the NDI database)	79,359	F 17.1%	NR	Suicide risk	HR 1.45 [95%CI 1.16–1.81], *p* < 0.001; HR 1.16 [95%CI 0.92–1.44], *p =* 0.09	No significant suicide risk when correcting for psychiatric conditions
Jimenez-Rodríguez et al. (29)	2014	Spain	Cross-sectional, case-control study	44	F 93.2%	54.5 ± 12.7	Active and passive SI, suicide risk	18 (41%) for passive SI, 6 (13.6%) for active SI, 36 (81.8%) for risk of suicide, OR 26.89 [95%CI 5.72–126.42] for SI, OR 48.0 [12.93–178.21] for suicide risk	Higher SI and suicide risk with respect to controls
Calandre et al. (30)	2015	Spain	Cross-sectional study	373	F 94.6%	49 ± 8.6	SI	148 (39.7%) for passive SI, 31 (8.3%) for active SI	Higher SI rate among FM patients
Triñanes et al. (31)	2015	Spain	Cross-sectional study	117	F 100%	49.09 ± 9.26 [22–80]	SI (generally passive)	38 (32.5%) for SI	Higher SI rate among FM patients with respect to general population
Lan et al. (32)	2016	Taiwan	Retrospective nationwide database-based, matched case-control study (data drawn from the Longitudinal Health Insurance Database, a subset of the NHIRD)	95,150	F 58.4%	45.8 ± 17.2	Suicide event	347 suicide events (4.16/10,000 person-years). Crude HR 1.58 [95%CI 1.38–1.83] and adjusted HR 1.38 [95%CI 1.19–1.60] for suicide event	Increased suicidal behaviors among FM patients, with an overall mild-to-moderate risk of suicide events
Lafuente-Castro et al. (33)	2018	Spain	Case-control study	38 without SI, 15 with SI	F 96.0%	52 ± 8.2	SI, suicide risk	OR 19.054 [95%CI 2.405–150.935] for SI, OR 30.055 [95%CI 10.247–100.269] for suicide risk	Increased suicidal behaviors among FM patients
McKernan et al. (34)	2018	USA	Retrospective database-based	8,879	F 90.9%	57^a^	SI, SA	96 for SI, 34 for SA	Increased suicidal behaviors among FM patients

*CI, confidence interval; F, female; FM, fibromyalgia; HR, hazard ratio; LBP, low back pain; NDB, National Data Bank for Rheumatic Diseases; NDI, National Death Index; NHIRD, National Health Insurance Research Database; NR, not reported; OR, odds ratio; RA, rheumatoid arthritis; SA, suicide attempt; SB, suicidal behaviour; SI, suicide ideation; SMR, standardized mortality ratio; SRS, Suicide Risk Scale; VA, Veterans Affairs*.

a *Median*.

The majority of studies demonstrated an increased risk of suicidal behavior amongst fibromyalgia patients, however these findings were not replicated in three studies included in this meta-analysis (20, 23, 28). When evaluating the determinants of suicidality among fibromyalgia patients, poor sleep quality was identified as a predictor of suicidality by several studies (26, 29–31). Triñanes and co-workers (31) and other studies, demonstrated depression and in particular, cognitive depression symptoms such as those identified by the Beck Depression Inventory (BDI) Self-Blame sub-scale, to be closely related to suicidal ideation. Additional risk factors reported were age (32), employment status (26), occupation (32), disease severity (26), obesity (34), psychological traits such as perceived burdensomeness, thwarted belongingness and poor marital adjustment (33) and drug dependence (34).

### Study Appraisal

Concerning the methodological quality of the included studies, one (20), five (24, 26, 29, 31, 33), three (25, 27, 30), one (23) and three (28, 32, 34) were deemed of fair, fair-to-moderate, moderate, moderate-to-high and high quality, respectively.

### Meta-Analysis

Rates of suicidal ideation were derived from 4 studies and ranged from 1.08% in the study by McKernan et al. (34) to 54.6% in the study by Jimenez-Rodríguez and co-authors (29). In particular, passive suicidal ideation ranged from 39.7 to 41.0%, whereas active suicidal ideation ranged from 8.3% in the study by Calandra and collaborators (30) to 13.6% in the study by Jimenez-Rodríguez et al. (30). Pooled rate for suicidal ideation, active and passive suicidal ideation resulted 29.57% [95%CI 1.84–72.07], 39.86% [95%CI 35.14–44.72], and 9.00% [95%CI 6.44–12.16], respectively. In the last 2 cases (active and passive suicidal ideation), heterogeneity was low and statistically not significant and, therefore, fixed-effect model was applied, whereas for the overall suicidal ideation rate due to the significant amount of heterogeneity (*Q* = 819.97, *I*^2^ = 99.63% [95%CI 99.51–99.73], *p* < 0.001), the random-effect model was performed. OR was derived from 3 studies and ranged from 2.34 ([95%CI 1.49–3.66], *z* = 3.71, *p* < 0.001) in the study by Ratcliffe and co-workers (23) to 26.89 ([95%CI 5.72–126.42], *z* = 4.17, *p* < 0.001) in the study by Jimenez-Rodríguez and co-authors (29). Due to the highly statistically significant heterogeneity (*I*^2^ = 83.27), the random-effect model was applied. Pooled OR resulted 9.12 ([95%CI 1.42–58.77], *z* = 2.33, *p* = 0.020), when not adjusting for psychiatric co-morbidities. However, due to the presence of publication bias, the “true” ES resulted 2.34 ([95%CI 0.48–11.52], not statistically significant). Correcting for confounding factors, the pooled OR resulted 7.60 ([95%CI 0.72–80.17], *z* = 1.69, *p* = 0.092). Once again, evidence of publication bias could be detected. Performing the trim-and-fill analysis, the “true” OR yielded 1.21 ([95%CI 0.15–9.83], not statistically significant). Interestingly, it was not statistically significant in both cases.

For suicidal attempt, rates were derived from 2 studies and ranged from 16.7 [95%CI 11.5–22.9] in the study by Calandre and co-authors (26) to 0.4 [95%CI 0.3–0.5] in the study by McKernan and co-workers (34). Due to the highly statistically significant heterogeneity (*Q* = 92.27, *I*^2^ = 98.9 [95%CI 97.8–99.5], *p* < 0.001), the random-effect model was performed. The overall pooled ES resulted 5.69 [95%CI 1.26–31.34]. OR resulted 3.12 [95%CI 1.37–7.12] as computed in the study by Ratcliffe and colleagues (23). However, statistical significance was lost, when adjusting for psychiatric co-morbidities (OR 1.27 [95%CI 0.54–3.01]).

For suicide risk, OR ranged from 30.06 ([95%CI 10.25–100.27], *z* = 5.85, *p* < 0.001) in the study by Lafuente-Castro et al. (33) to 48.0 ([95%CI 12.93–178.21], *z* = 5.78, *p* < 0.001) in the study by Jimenez-Rodríguez et al. (29). Pooled OR resulted 36.77 ([95%CI 15.55–96.94], *z* = 8.21, *p* < 0.001). In terms of HR, suicide risk resulted 1.45 ([95%CI 1.16–1.81], *p* < 0.001) as computed in the study by Ilgen and collaborators (28). However, statistical significance was lost when adjusting for psychiatric conditions (HR 1.16 [95%CI 0.92–1.44], *p* = 0.09).

Concerning suicide event, HR resulted 1.38 [95%CI 1.17–1.71], adjusting for psychiatric conditions, as computed in the study by Lan et al. (32). Incidence of suicide events among fibromyalgia patients was 31/100,000 person/years, as computed in the study by Cheng and co-workers (24). Mortality OR resulted 3.31 [95%CI 2.15–5.11] with respect to general population, as reported in the study by Wolfe and co-authors (27). Standardized mortality rate was 10.5 [95%CI 4.5–20.7], as reported by Dreyer and colleagues (25). All these values were not adjusted for underlying psychiatric conditions (24, 25, 27).

**Figure 2:**
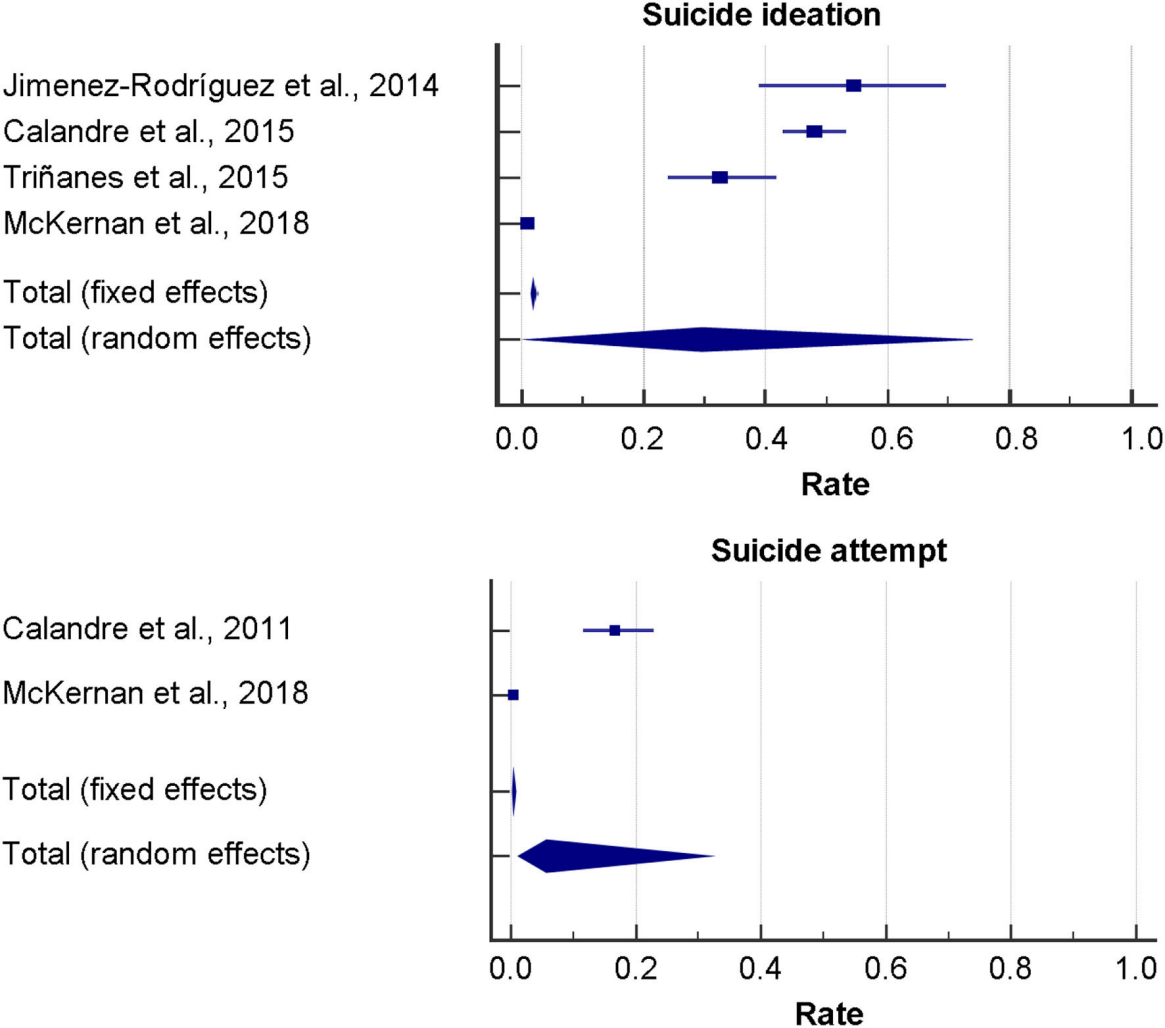
Forest plots of suicide ideation and attempt rates among fibromyalgia patients.

Relevant forest plots are shown in Figure 2 and tabulated in Table 3.

**Table 3:** The main findings of the systematic review and meta-analysis on suicidality among fibromyalgia patients.

**Variable**	**Results**
Suicide ideation	Overall suicide ideation rate 29.57% [95%CI 1.84–72.07]; 1.08–54.6%
	Passive suicide ideation rate 39.86% [95%CI 35.14–44.72]; 39.7–41.0%
	Active suicide ideation rate 9.00% [95%CI 6.44–12.16]; 8.3–13.6%
	OR 9.12 [95%CI 1.42–58.77]; 2.34 [95%CI 1.49–3.66]−26.89 [95%CI 5.72–126.42]
	OR 7.60 [95%CI 0.72–80.17] adjusted for psychiatric conditions
Suicide attempt	Rate 5.69% [95%CI 1.26–31.34]; 0.38–16.67%
	OR 3.12 [95%CI 1.37–7.12]
	OR 1.27 [95%CI 0.54–3.01] adjusted for psychiatric conditions
Suicide risk	OR 36.77 [95%CI 15.55–96.94]; 30.06 [95%CI 10.25–100.27]−48.0 [95%CI 12.93–178.21]
	HR 1.45 [95%CI 1.16–1.81]
	HR 1.16 [95%CI 0.92–1.44] adjusted for psychiatric conditions
Suicide event	HR 1.38 [95%CI 1.17–1.71]
	Incidence 31/100,000 person/years
	Mortality OR 3.31 [95%CI 2.15–5.11]
	SMR 10.5 [95%CI 4.5–20.7]

*CI, confidence interval; HR, hazard-ratio; OR, odds-ratio; SMR, standardized mortality rate*.

## Discussion

Fibromyalgia is a chronic pain syndrome closely associated with psychiatric comorbidities, sleep disturbances and fatigue, which all contribute to a detrimental effect on quality of life. The close link between fibromyalgia and depression, a known risk factor for suicide, has supported the theory that fibromyalgia patients may have a higher risk of suicidal ideation and behavior, however, contrasting findings have been reported in the literature. The findings from our large-scale and rigorously conducted systematic review and meta-analysis indicate that while fibromyalgia patients do have an overall increased risk of suicidal behavior and attempts, this association may be due to the confounding presence of chronic and psychiatric comorbidities.

Several studies have demonstrated the relationship between chronic pain syndromes and suicide. Interestingly, it appears that while the physical characteristics of pain, such as intensity and type, are of importance, it is rather the emotional, psychological interpretation of pain that significantly impacts and drive suicidality (35, 36). In the study by Calandre et al. (30) included in this review, physical pain was only weakly associated with suicidal ideation in fibromyalgia patients. In another included study, which compared suicidal ideation and attempts between fibromyalgia patients and patients with lower back pain, the risk was greater for fibromyalgia patients, despite similar mean pain intensity scores for both groups (29). This suggests that personality traits and behaviors commonly associated with fibromyalgia, such as neuroticism and catastrophizing, predispose fibromyalgia patients to distorted pain perception and further amplifies their suicidality risk (17, 36, 37).

When evaluating the relationship between fibromyalgia and suicidality, it is important to note the association between fibromyalgia with several well-known suicidal risk factors, such as female gender, psychiatric comorbidity and sleep disturbance. Additionally, it is important to address the significant impact of demographic and socioeconomic factors on suicidality, such as marital and employment status, which are also known to be lower in fibromyalgia patients (33). While conducting this systematic review, we found that disease severity, employment status and comorbidities such as obesity, drug dependence, anxiety, poor sleep, global mental health and in particular, depression, were all important negative factors which contributed to the overall risk of suicide in fibromyalgia patients. As noted before, when the confounding effect of the comorbidity factors was evaluated, fibromyalgia was not shown to have an independent association with suicidal ideation and attempts. Thus, it may be inferred that it is not the chronic condition of fibromyalgia *alone* which leads to a higher risk of suicidality, yet rather the impact of the comorbidities, mainly psychiatric, which drives the increased risk. However, it remains to be seen whether the presence of fibromyalgia may augment the suicidal risk effect conferred by psychiatric illness, mainly depression. It has been hypothesized that the fibromyalgia syndrome is the results of a neurogenic neuroinflammatory reaction in which physical and psychological trauma trigger CNS sensitization and subsequently, widespread pain and mood disorders. Major depressive disorder has also been recently linked to immune system activation in the CNS, which raises the possibility of a combined detrimental neuroinflammatory impact leading to an increased risk of suicidality in fibromyalgia patients with depression (38). Another theory indicates the important role of serotonergic neurotransmission, which has been demonstrated to be decreased in fibromyalgia patients and independently associated with an increased risk of suicide (32, 39, 40).

Although thorough, this meta-analysis demonstrates the paucity of information existing in the literature regarding suicidality amongst fibromyalgia patients. Furthermore, the low quality of evidence should be noted. With the only exception of the investigation conducted by Dreyer et al. (26), the majority of the studies included in this meta-analysis were retrospective and/or cross-sectional, and as such, impeded our ability to determine a causal association between suicidality and fibromyalgia. Moreover, despite the critical importance of taking psychological comorbidities into account, only five studies adjusted for co-morbid chronic conditions and only six investigations adjusted for co-occurring psychiatric disorders. Nevertheless, this systematic review and meta-analysis sheds light upon the important confounding effect of said comorbidities when assessing the relationship between fibromyalgia and suicidal ideation and behaviors.

Our study's major strength relies on its methodological rigor, transparency and reproducibility as a systematic review and meta-analysis conducted in accordance with the PRISMA guidelines, in addition to the extensive literature search, cross-checking and cross-referencing we conducted. However, our study suffers from several limitations, which should be properly recognized. The major shortcoming was the small number of studies retained for each outcome, which precluded the possibility of running meta-regressions based on different co-factors and co-variates, such as study design, diagnostic criteria utilized for fibromyalgia and suicidality/suicidal behaviors, use of validated tools or self-report questionnaires, and further stratifying according to underlying chronic and/or psychiatric co-morbidities. Moreover, it is noteworthy that the largest sample size included in the present systematic review and meta-analysis (34) could not be adequately controlled for diagnostic criteria and quality. Another limitation was the high amount of heterogeneity and the presence of publication bias, which calls for caution when interpreting and generalizing the present findings. Nevertheless, our study demonstrates the importance of further elucidating and determining suicidality risk factors. Given the complex, non-linear, multi-factorial etiopathogenesis of suicidal behaviors, longitudinal studies are warranted. Also, the use of structural equation modeling could be particularly effective, considering the reciprocal relationship among the variables, in order to avoid “ceiling effects.”

## Conclusions

The present systematic review and meta-analysis has practical implications for rheumatologists, who should be aware of the clinically relevant suicide risk among fibromyalgia patients. We found that fibromyalgia is associated with an increased rate of suicidality, in terms of ideation, attempt, risk and events, warranting a pre-emptive screening of their mental health status. However, in some cases, after adjusting for psychiatric conditions, the threshold of statistical significance was not achieved. Given the few studies available, the high amount of heterogeneity and the evidence of publications bias, further high-quality studies should be conducted, with a focus on longitudinal studies.

## Data Availability Statement

The original contributions presented in the study are included in the article/Supplementary Material, further inquiries can be directed to the corresponding authors.

## Author Contributions

MA, NB, DM, and HA: conceptualization. AW, AK, DM, YY, DA, and HA: data curation. NB: formal analysis and methodology. MA and HA: supervision. MA, AK, YY, and HA: writing—original draft. MA, NB, AK, YY, AW, DM, DA, and HA: writing—review and editing. All authors contributed to the article and approved the submitted version.

## Conflict of Interest

The authors declare that the research was conducted in the absence of any commercial or financial relationships that could be construed as a potential conflict of interest.

## Publisher's Note

All claims expressed in this article are solely those of the authors and do not necessarily represent those of their affiliated organizations, or those of the publisher, the editors and the reviewers. Any product that may be evaluated in this article, or claim that may be made by its manufacturer, is not guaranteed or endorsed by the publisher.
