# Supplementary material for: Suicidal Behavior in Fibromyalgia Patients: Rates and Determinants of Suicide Ideation, Risk, Suicide, and Suicidal Attempts—A Systematic Review of the Literature and Meta-Analysis of Over 390,000 Fibromyalgia Patients
Source: Front Psychiatry. 2021 Nov 19;12:629417. doi: 10.3389/fpsyt.2021.629417 (PMC8640182; doi:10.3389/fpsyt.2021.629417)
Supplement: Supplementary file 1 [file Data_Sheet_1.docx]

**Supplementary material.**

**Supplementary Table 1. Search strategy adopted in the present systematic review and meta-analysis.**

| **Search strategy item** | **Details** |
| --- | --- |
| Keywords | Fibromyalgia AND (suicide OR “suicidal ideation” OR “suicidal thoughts” OR “risk of suicide” OR “suicide risk” OR “suicide attempt” OR “suicidal behaviour” OR suicidality OR “death wish” OR “death desire”) |
| Databases | PubMed/MEDLINE, Scopus, ISI/WoS, Google Scholar, DOAJ |
| Inclusion criteria | P: fibromyalgia patients with fibromyalgia as the main diagnosis  I: suicidal behaviour (suicide ideation, attempt or event)  C: any comparator (severity and co-morbidity of fibromyalgia)  O: prevalence/incidence rate, determinants of suicidal behaviour  S: any type of observational study (cross-sectional, retrospective, case-control, etc.) |
| Exclusion criteria | Studies in which fibromyalgia is a co-morbidity and not the main diagnosis  S: letter to editor, editorial, commentary, expert opinion, clinical case report or series; review article; interventional study |
| Time filter | None (from inception) |
| Language filter | None (any language) |
| Target journals | Arthritis Rheum; Neuropsychiatr Dis Treat; Pain Pract; Rheumatol Int; Rheumatology (Oxford) |

***Abbreviations***: DOAJ (Directory of Open Access Journals); WoS (Web of Science).

**Supplementary Table 2. Relevant data abstracted from included studies.**

| **Data abstracted** | **Details** |
| --- | --- |
| Reference | First author of the study |
| Study publication year | Year in which the study was published |
| Country | Country in which the study was performed |
| Study design | Cross-sectional, retrospective database-based, matched case-control study |
| Sample size | Number of recruited fibromyalgia patients |
| Characteristics of the recruited sample  Age  Female percentage  Educational level  Employment status  Marital status  Disease activity  Co-morbidities  Psychiatric co-morbidities  Treatment | Age expressed as mean±standard deviation or median; age range (in years)  Number of female patients expressed as percentage  Number of patients who completed primary education expressed as percentage  Unemployed expressed as percentage  Number of married patients expressed as percentage  Expressed in years  Expressed as percentage  Expressed as percentage  Drugs administered to patients |
| Fibromyalgia diagnostic criteria | Diagnostic criteria used for diagnosing fibromyalgia or retrieving fibromyalgia patients from computerized databases (including the 1990 and 2010 ACR criteria, and the ICD9/ICD9-CM) |
| Instrument for assessing suicidal behaviour | Item 9 of the Beck’s Depression Inventory (0 points “I don't have any thoughts of killing myself”; 1 point “I have thoughts of killing myself, but I would not carry them out”; 2 points “I would like to kill myself”; 3 points “I would kill myself if I had the chance”); the Plutchik Suicide Risk Scale (PSRS), a 15-item questionnaire; the 26-item Suicide Risk Scale (SRS), a 26-item questionnaire; ICD-8/ICD-10; *ad hoc* questions (“Have you ever seriously thought about committing suicide or taking your own life in the last 12 months? Have you ever attempted suicide or tried to take your own life?” / “Did the patient attempt suicide? If affirmative, how many times? Which was the employed method? Did she/he require attendance in the emergency room and/or require hospitalization for ≥24 h?”) |
| Study quality | Methodological quality critically appraised using the Newcastle-Ottawa scale |

|  |  | | |  | | **Supplementary Table 3. Basic characteristics of included studies.** | | | | | | | | | | | | | | | | | | |  |
| --- | --- | --- | --- | --- | --- | --- | --- | --- | --- | --- | --- | --- | --- | --- | --- | --- | --- | --- | --- | --- | --- | --- | --- | --- | --- |
| **Authors** | | **Year** | **Country** | | **Study design** | | **Instrument for screening suicide** | **Sample size** | **FM diagnostic criteria** | **Gender** | **Age** | **Educational level** | **Employment status** | **Marital status** | **Disease activity** | **Co-morbidities** | **Psychiatric co-morbidities** | **Treatment** | **Correction for co-morbid chronic conditions** | **Correction for co-morbid mental disorders** | **Suicidal variable studied** | **Epidemiological findings** | **Determinants** | **Main findings** | **Study quality** |
| Amir et al., [22] | | 2000 | Israel | | Cross-sectional, case-control study | | SRS (Cronbach’s alpha 0.88) | 51 outpatients randomly chosen from 600 | 1990 ACR | F 100% | 48.96±8.41 | 10.5±3.6 years | NR | 72% | NR | NR | NR | NR | No | No | Suicide risk | 44.5±8.4 score | NR | No differences in terms of suicide risk with general population and other patients (RA, LBP) | Fair |
| Ratcliffe et al., [23] | | 2008 | Canada | | Retrospective database-based study (data drawn from the Canadian Community Health Survey Cycle 1.2 file) study | | *Ad hoc* questions conducted in person or by telephone | 595 | *Ad hoc* question (“Do you suffer from any long-term conditions, since 6 months or more, that had been diagnosed by a health professional?”) with acceptable to good concordance between self-report and medical record measures | NR | NR | NR | NR | NR | NR | 82.7% with a comorbid chronic pain condition (36.7% migraine, 53.9% arthritis and 52.5% back problems) | NR | NR | Yes | Yes | SI, SA | OR ranging from 1.21 [95%CI 0.68-2.18] to 2.34 [95%CI 1.49-3.66] for SI,  OR ranging from 1.27 [95%CI 0.54-3.01] to 3.12 [95%CI 1.37-7.12] for SA | Chronic pain and co-morbidities | No significant SI and SA OR after adjusting for psychiatric conditions and/or other co-morbid chronic pan conditions | Moderate-to-high |
| Cheng et al., [24] | | 2009 | USA | | Retrospective database-based study | | NR | 199,739 | NR | NR | NR | NR | NR | NR | NR | NR | NR | NR | No | No | Suicide risk | Incidence 31/100,000 person/years for SB  OR 3.5 for SB | NR | Increased suicide risk among FM patients | Fair-to-moderate |
| Dreyer et al., [25] | | 2010 | Denmark | | Retrospective-prospective database-based study (data drawn from the hospital database and from the Danish Mortality Register) | | ICD-8 (from 1969 to 1983), ICD-10 (from 1983 on) | 1,269 (1,353 out of an initial list of 1,361) | 1990 ACR, clinical interview and physical examination | F 93.2% | 58.2% in the range 30-49 years | NR | NR | NR | NR | Sjögren's syndrome 0.2%, RA 0.4%, PsA 0.1%, polymyositis 0.1%, AS 0.1% | NR | NR | No | No | Suicide event | 8 suicide events (SMR 10.5 [95%CI 4.5-20.7] for all patients,  4 suicide events (SMR 6.5 [95%CI 1.8-16.7] for confirmed FM patients,  2 suicide events (SMR 19.6 [95%CI 2.2-70.8] for possible FM patients | NR | Increased suicide risk at the time of diagnosis until 5 years after the diagnosis | Moderate |
| Calandre et al., [26] | | 2011 | Spain | | Cross-sectional study | | *Ad hoc* questions *via* surveys and PSRS | 180 out of 795 (completion rate of 22.6%) | FIQR | F 97.8% | 51±8.5 [22-74] | Primary school 56.6%, 23.4% secondary school, university 12.0%, no school 8% | 13.6% unemployed, 27.7% on leave, 12.4% retired, 27.1% work only at home | 78.1% | 13.1±8.5 [1-54] (7.1±4.8 since diagnosis, [1-23]) | 2.73±1.6 [0-8] (30.6% arthrosis, 17.8% disk herniation, 12.8% osteoporosis/osteopenia, 5.0% CTS, 11.1% thyroid disorders, 10.6% IBS/Crohn’s disease, 8.3% headache, 7.2% allergies, 6.7% asthma, 6.7% hypertension, 6.7% hypercholesterolemia, 5.6% CFS) | 1.19±0.5 [0-3] (13.3% depression, 4.4% anxiety, 3.3% mixed anxiety-depressive disorder, 2.2% insomnia, 1.1% mental disorder NOS, 0.6% agoraphobia and social phobia) | NR | No | No | SA | 30 (16.7%) for SA (21 (70%) by drug poisoning); 20 (66.7%) one SA, 5 (16.7%) two SA and 5 (16.7%) reported three SA | Employment status and disease severity (pain symptoms), sleep disorders, depression, anxiety | Higher SA rate with respect to general population | Fair-to-moderate |
| Wolfe et al., [27] | | 2011 | USA | | Retrospective database-based study (data drawn from the Wichita nonparticipant group file and from the NDB) | | Suicide event | 8,186 | Experienced rheumatologists, 1990 ACR, 2010 ACR, fibromyalgianess scale | F 93.9% | 50.5±12.4 | High school graduate 93.7%, college graduated 29.5% | NR | 68.4% | NR | NR | NR | NR | No | No | Suicide risk | OR 3.31 [95%CI 2.15-5.11] with respect to general population | NR | Increased suicide risk | Moderate |
| Ilgen et al., [28] | | 2013 | USA | | Retrospective database-based study (data drawn from the VA National Patient Care Database and the NDI database) | | Suicide event | 79,359 | ICD-9-CM | F 17.1% | NR | NR | NR | NR | NR | NR | NR | NR | Yes | Yes | Suicide risk | HR 1.45 [95%CI 1.16-1.81], p<0.001; HR 1.16 [95%CI 0.92-1.44], p=0.09 | NR | No significant suicide risk when correcting for psychiatric conditions | High |
| Jimenez-Rodríguez et al., [29] | | 2014 | Spain | | Cross-sectional, case-control study | | BDI (Cronbach’s alpha 0.83) and PSRS (Cronbach’s alpha 0.9) | 44 | 1990 ACR | F 93.2% | 54.5±12.7 | No school/primary school 38.6%, secondary school/university 18.2% | Unemployed 9.1%, retired 9.1%, work only at home, 4.5% on leave | 38.6% | NR | NR | 86.4% sleep disorders, 91% depression; no other mental disorders | NR | No | Yes | Active and passive SI, suicide risk | 18 (41%) for passive SI,  6 (13.6%) for active SI,  36 (81.8%) for risk of suicide,  OR 26.89 [95%CI 5.72–126.42] for SI,  OR 48.0 [12.93–178.21] for suicide risk | Sleep disorders, depression, self-perceived health-related quality of life; in the predictive model, only depression associated with SI (OR 1.183 [95%CI 1.068–1.311], p=0.001) and with suicide risk (OR 1.159, 95%CI 1.046–1.283, p=0.005) | Higher SI and suicide risk with respect to controls | Fair-to-moderate |
| Calandre et al., [30] | | 2015 | Spain | | Cross-sectional study | | BDI | 373 | 1990 ACR, FIQ | F 94.6% | 49±8.6 | NR | NR | NR | 3.8±3.8 [1-33] | 4.8±2.1 [0-13] | NR | NR | Yes | Yes | SI | 148 (39.7%) for passive SI  31 (8.3%) for active SI | Depression, anxiety, sleep quality, and global mental health | Higher SI rate among FM patients | Moderate |
| Triñanes et al., [31] | | 2015 | Spain | | Cross-sectional study | | BDI | 117 | 1990 ACR, clinical interview, physical examination and FIQ | F 100% | 49.09±9.26 [22-80] | Primary school 61.5%, high school 20.5% , university 17.1%, never studied 0.9% | 62.4% full-time job, 16.7% on leave or with work disability, 5.60% unemployed, 3.7% never worked, 13.9% retired | 86.3% | 8.25±6.21 | 0% (co-morbid chronic condition free) | 0% (co-morbid psychiatric condition free) | 18.8% analgesic medication, 11.1% anxiolytic drugs, 9.4% antidepressants, 2.6% anti-convulsants, 6% analgesics + antidepressants, 14.5% anxiolytics + antidepressants, 17.9% other drugs, 19.7% no drugs | NA | NA | SI (generally passive) | 38 (32.5%) for SI | Depression (self-blame), impact of the disease, sleep disorders and anxiety | Higher SI rate among FM patients with respect to general population | Fair-to-moderate |
| Lan et al., [32] | | 2016 | Taiwan | | Retrospective nationwide database-based, matched case-control study (data drawn from the Longitudinal Health Insurance Database, a subset of the NHIRD) | | Suicide event (completed suicide, suicide attempt, nonsuicidal self-inflicted injury) | 95,150 | ICD 9-CM | F 58.4% | 45.8±17.2 | NR | 50.6% office workers, 40.6% manual workers, 8.78% others | NR | NR | NR | 4.27% depression, 9.88% anxiety, 19.8% sleep disorders, 8.83% irritable bowel syndrome, 5.16% headache | 45.2% NSAIDs | Yes | Yes | Suicide event | 347 suicide events (4.16/10,000 person-years)  Crude HR 1.58 [95%CI 1.38-1.83] and adjusted HR 1.38 [95%CI 1.19–1.60] for suicide event | Age (<65 years), job (office workers), income, co-morbidities (FM patients and co-morbidities having a HR of 1.51-8.23 with respect to FM- and co-morbidities-free patients, much higher than the HR of 1.33-1.69 of primary FM patients) | Increased suicidal behaviours among FM patients, with an overall mild-to-moderate risk of suicide events | High |
| Lafuente-Castro et al., [33] | | 2018 | Spain | | Case-control study | | PSRS | 38 without SI, 15 with SI | 1990 ACR | F 96.0% | 52±8.2 | Primary school 54.7%, secondary school 28.3%, university 16.9% | Unemployed 13.2%, work at home 11.3%, temporary leave 11.3%, permanent leave 20.8%, retired 13.2% | 69.8% | NR | NR | 0% (chronic conditions free) | 0% (psychiatric disorders free) | NA | NA | SI, suicide risk | OR 19.054 [95%CI 2.405–150.935] for SI,  OR 30.055 [95%CI 10.247–100.269] for suicide risk | Perceived burdensomeness, thwarted belongingness and poor marital adjustment | Increased suicidal behaviours among FM patients | Fair-to-moderate |
| McKernan et al., [34] | | 2018 | USA | | Retrospective database-based | | Machine learning techniques, utilizing literature review, theoretical models and frameworks, clinical expertise | 8,879 | PheKB validated phenotype combining diagnostic codes (from ICD-9), clinical expertise and textual information | F 90.9% | 57^a^ | NR | NR | NR | NR | NR | NR | NR | Yes | Yes | SI, SA | 96 for SI  34 for SA | Fatigue (OR=1.29, 95%CI 1.25-1.32), dizziness (OR=1.25, 95%CI 1.22-1.28), weakness (OR=1.17, 95%CI 1.15-1.19), obesity (OR=1.18, 95%CI 1.10-1.27) and drug dependence (OR=1.15, 95%CI 1.12-1.18), co-morbidities | Increased suicidal behaviours among FM patients | High |

***Abbreviations***: ACR (American College of Rheumatology); AS (ankylosing spondylitis); CFS (chronic fatigue syndrome); CI (confidence interval); CTS (carpal tunnel syndrome); F (female); FIQ (Fibromyalgia Impact Questionnaire); FIQR (Fibromyalgia Impact Questionnaire Revised); FM (fibromyalgia); HR (hazard ratio); IBS (irritable bowel syndrome); ICD-8 (International Classification of Diseases – Eighth revision); ICD-9 (International Classification of Diseases – Ninth revision); ICD-10 (International Classification of Diseases – Tenth revision); LBP (low back pain); NA (not applicable); NDB (National Data Bank for Rheumatic Diseases); NDI (National Death Index); NHIRD (National Health Insurance Research Database); NOS (not otherwise specified); NR (not reported); OR (odds ratio); PsA (psoriatic arthritis); RA (rheumatoid arthritis); SA (suicide attempt); SI (suicide ideation); SMR (standardized mortality ratio); SRS (Suicide Risk Scale); VA (Veterans Affairs).

^a^median
